# Reduction of inequalities in health: assessing evidence-based tools

**DOI:** 10.1186/1475-9276-5-11

**Published:** 2006-09-27

**Authors:** Peter Tugwell, Annette O'Connor, Neil Andersson, Sharmila Mhatre, Elizabeth Kristjansson, Mary Jane Jacobsen, Vivian Robinson, Jan Hatcher-Roberts, Beverley Shea, Daniel Francis, Jil Beardmore, George A Wells, Joe Losos

**Affiliations:** 1 Institute of Population Health, University of Ottawa, 1 Stewart Street, Ottawa, Ontario, K1N 6N5, Canada; 2 Ottawa Health Research Institute, Ottawa, Canada; 3 Community Interventions and Epidemiological Technologies (CIET Canada), Ottawa, Canada; 4 International Development Research Centre, Ottawa, Canada; 5 Canadian Society for International Health, Ottawa, Canada; 6 VU University Medical Centre, Amsterdam, The Netherlands; 7 Department of Epidemiology and Community Medicine, University of Ottawa, Ottawa, Canada

## Abstract

**Background:**

The reduction of health inequalities is a focus of many national and international health organisations. The need for pragmatic evidence-based approaches has led to the development of a number of evidence-based equity initiatives. This paper describes a new program that focuses upon evidence- based tools, which are useful for policy initiatives that reduce inequities.

**Methods:**

This paper is based on a presentation that was given at the "Regional Consultation on Policy Tools: Equity in Population Health Reports," held in Toronto, Canada in June 2002.

**Results:**

Five assessment tools were presented. 1. A database of systematic reviews on the effects of educational, legal, social, and health interventions to reduce unfair inequalities is being established through the Cochrane and Campbell Collaborations. 2 Decision aids and shared decision making can be facilitated in disadvantaged groups by 'health coaches' to help people become better decision makers, negotiators, and navigators of the health system; a pilot study in Chile has provided proof of this concept. 3. The CIET Cycle: Combining adapted cluster survey techniques with qualitative methods, CIET's population based applications support evidence-based decision making at local and national levels. The CIET map generates maps directly from survey or routine institutional data, to be used as evidence-based decisions aids. Complex data can be displayed attractively, providing an important tool for studying and comparing health indicators among and between different populations. 4. The Ottawa Equity Gauge is applying the Global Equity Gauge Alliance framework to an industrialised country setting. 5 The Needs-Based Health Assessment Toolkit, established to assemble information on which clinical and health policy decisions can be based, is being expanded to ensure a focus on distribution and average health indicators.

**Conclusion:**

Evidence-based planning tools have much to offer the goal of equitable health development.

## Background

Social gradients in health have been documented in most countries throughout the world [1]. These socio-economic inequalities are demonstrated by uneven patterns of disease, injuries, and health behaviours across socio-economic groups. Inequalities are termed inequities when these inequalities are deemed to be unfair and avoidable. They represent needless human suffering and lost productivity; they also have significant consequences for the economy and for social order and justice [2].

Health inequities have long been a focus of national and international health organizations. For decades, much of the concern about health equity found expression in the movement for primary health care (PHC), launched at the International Conference on Primary Health Care at Alma Ata in 1978. Despite the explicit commitment to PHC made by the world's governments in the Alma-Ata Declaration, the goal of 'Health for All' is arguably as distant now as it was then. Indeed, evidence exists of a widening gap in health gains between poor and rich countries and between the poor and rich within countries. At the 1998 Almaty meeting, commemorating the 20^th ^anniversary of the Alma Ata declaration, it was agreed that a more pragmatic evidence-based approach was needed to deal with health inequity. It is notable that the International Society for Equity in Health highlights an actionable definition of equity (in policy and actions) as "Active policy decisions and programmatic actions directed at improving equity in health or in reducing or eliminating inequalities in health."[3]

Evidence-based approaches have become central to many equity-oriented initiatives. For example, in the 1980s and 90s, UNICEF promoted what it called the Triple-A approach. The concept was a reiterative feedback loop (assessment-analysis-action) that targeted inequities underlying child malnutrition. It recognised that conditions varied from place to place and hence approaches to change conditions must also vary [4]. This concept was very clear about the need for reiteration, building on the achievements of each cycle of assessment, analysis and action.

Major international health donors also adapted evidence-based approaches to decreasing inequities. In the latter half of the 1990s, several United Nations organisations began employing rights-based programming, systematizing the allocation of resources to advocate in favour of the least advantaged. Furthermore, Results-Based Management (RBM) was promoted by the World Bank (often using CIET methods), with CIDA playing a leading role in embracing the reiterative use of evidence and measurement.

While measuring the severity and extent of inequities has gradually become more common, it is apparent that evidence must also be used to help identify interventions able to deal with inequalities [5]. In a 1999 consultation organized by the Rockefeller Foundation (in collaboration with the World Bank), participants agreed on a need for health equity research to shift the present static emphasis on measurement and analysis of health inequities towards dynamic identification and evaluation of policy measures [5]. The Equity Gauge initiative is intended as one such strategy that links measurement and analysis with the identification and evaluation of inequity reducing policies and actions [6].

Policy-level changes are very important in reducing health inequities. Priority-setting by policy-makers is affected by the values and interests of individuals in decision-making positions. While the rhetoric of equity is very well developed, there is often a failure to set equity-oriented objectives and action plans. This may not only be due to a lack of will on the part of policy makers but also because of the many barriers that hinder priority setting and planning. These barriers include a lack of skills for pro-equity planning, a lack of supportive institutional structures and processes, a lack of sophisticated understanding of what equity does and does not require, a lack of the intersectoral cooperation and unity (often necessary for real achievements), and a lack of incentives for achieving goals. In many cases, political barriers also exist, thereby hampering efforts to successfully plan and act on inequalities.

Planners have not always had access to appropriate data on social differentials and the needs and capacities of people to improve them. The translation of priorities into strategies and actions is at best based on the information available to planners, and the perceived costs and impacts of possible interventions. Data available in most countries come from institutions- the service users- rather than from the communities that governments are meant to represent and serve. Routine institutional data seldom capture the complex realities of communities, let alone the differentiated realities of different segments of the population. Where the skills to do so exist, these can be complemented by surveys that allow a sharper focus on inequity.

Because health equity depends so much on deep-seated power issues, on economic and ideological constructs largely outside the reach of health planners, perhaps the contribution of development agencies is limited to making measurement and analysis more readily available. Although these tools cannot in themselves produce equity, where conditions are such that increased equity is possible, tools must be made available to draw attention to inequities and to help redirect resources to where they are most needed. This paper presents an assessment of five such tools with which members of the Centre for Global Health at the Institute for Population Health, University of Ottawa, have had experience (Table 1).

**Table 1:** Critical Analysis of Tools

	**Cochrane and Campbell Collaborations**	**Decision Aids, Shared Decision Making and the Health Coach Initiative**	**CIET cycles**	**Ottawa Equity Gauge**	**The Needs-Based Health Assessment Toolkit**
Audience	Clinical decision makers/practitioners/policy makers/health consumers	Health care consumers and clinicians providing decision support	Decision makers at provincial, regional and national levels	Local policy makers, community agencies, schools, and non-government organizations	Health professionals, policy makers and health system planners
Objectives	They aim to help people make well informed decisions about health care by preparing, maintaining and disseminating systematic reviews.	Prepare individuals for decision making: help them understand the probable benefits and risks of options, consider the value they place on the benefits and risks, & participate actively with their practitioners in deciding about options	Bring scientific research methods to local government and community levels; build the community voice into planning and good governance	To bridge the gap from evidence to action in reducing health inequalities	To assist in the efficient and effective allocation of health care resources
Strengths	The Cochrane Library now has over 2000 reviews providing high quality, up to date summaries of evidence obtained through a transparent process aimed at avoiding bias.	Improved decision making outcomes (see 'Success')	Representative, community-based cross-design combines qualitative and quantitative data; emphasis on training and capacity building; methods adapted for a wide variety of issues	Actions are based on the best-evidence of interventions	The toolkit is based on a systematic and comprehensive framework for assembling the information on which clinical and health policy decisions about technologies can be based. It is needs-based according to clinical and population health status needs, and therefore not "wants-based" nor driven by the vested interests of health professions, industry, or government
Limitations	Many estimates are of efficacy in ideal situations, not effectiveness in a community setting. Also, only limited numbers of less rigorous non-controlled studies are included.	Most decision aids are web-based which increases universal access, but may limit access for some groups	CIET methods are less useful for rare conditions (cancers, maternal mortality) than for common risk factors or outcomes. Methods require considerable epidemiological analysis skills.	The process of engaging such a diverse range of stakeholders has presented a number of difficulties	The toolkit provides only a selected set of tools. Users must decide whether these tools can be adapted to their own settings and needs.
Success	Examples where Cochrane has been used, including in the policy environment . For example, a patient directed handout containing information from a Cochrane review of antibiotics and acute otitis media changed prescription rates in a general practice population in south London.	Research shows that DA's: increase patient participation in decision making (without increasing anxiety); improve decision quality (improved knowledge, more realistic expectations, better match between values and choices, lower decisional conflict, fewer undecided, reduce uptake of options patients do not value.	Methods have been used in 48 countries worldwide since 1985; research topics have included corruption in public services, Aboriginal health, prenatal care, landmine awareness and health, and HIV/AIDS and sexual violence.	The Ottawa Equity Gauge iis only 5 years old, but has successfully conducted a study of food security which is being used for advocacy and has recently obtained support for a study of geographic inequalities in food security in Ottawa. There have however been a number of identified successes of the Equity Gauge strategy in other countries where it has been implemented. (McCoy et al 2003	The project's main activity has been dissemination, training and policy dialogue. It is expected that developmental impact will be more apparent in the future because there was and continues to be sustained institutional support for those health professionals who completed their fellowships in Ottawa with the WHO Collaborating Centre for Health Technology. The Tool Kit is incorporated into graduate curricula and course materials. Usability and usefulness for policy-makers is being assessed by focus groups with policy-makers at local and international conferences (2004–2005)
Challenges	Challenges facing the Cochrane Collaboration include its future sustainability, its ability to prioritize reviews, and to further influence consumers, practitioners and policy makers.	Integration into the process of care	Building epidemiology skills. Communicating evidence to decision makers in a way they can understand; logistics of fieldwork in difficult and sometimes dangerous conditions.	Building relationships with community, grass-roots groups and policy and decision-makers is challenging. Also, it is difficult to ensure that community needs remain the main driver of the Ottawa Equity Gauge	Ensuring that the toolkit is updated to reflect new tools available for Health Technology Assessment. Evaluation of the impact of the toolkit.

## Methods

This paper is based on the presentation "Assessing evidence-based tools for reducing Inequalities and Inequities," given at the "Regional Consultation on Policy Tools: Equity in Population Health Reports," held in Toronto in June 2002 in conjunction with the International Society for Equity and Health biannual conference. The audience, objectives, strengths, limitations and successes of five tools from this presentation were assessed based on descriptions of their purpose, randomized trials and observational evidence of their strengths and limitations and descriptions of success from the authors or from their annual reports. Conference participants were divided into small-group break-out sessions following this presentation to make recommendations on future research directions.

## Results

### 1. Cochrane and Campbell collaborations

The Cochrane [7] and Campbell [8] collaborations were established to prepare, maintain and promote access to systematic reviews thereby helping consumers, policy-makers and clinicians make well-informed decisions. The Cochrane Collaboration reviews studies of the effects of health and health care policies and the Campbell Collaboration reviews the studies of educational, legal and social interventions.

Most reviews present information on effectiveness in terms of averages, without providing any indication of the effectiveness of interventions stratified by socio-economic gradients. If inequalities are present, the recommendation typically carries out statistical adjustment to remove the effect.

Plans to identify interventions that improve the status of the poor and reduce health inequities through a series of systematic reviews are now underway within the Cochrane and Campbell Collaborations. A Cochrane Equity Field has successfully been registered to help deal with the methodological issues that arise from incorporating equity into systematic reviews. Equity gradients relevant to informing policy and decision makers on the effectiveness of interventions include not only socioeconomic gradients but also gender, race, workforce, rural-urban, education and social capital gradients. Examples include those impacting on infections such as peer support for HIV, insecticide treated bed-nets, direct observation of therapy for TB, risk factors such as school nutrition programs, smoking prevention, and chronic conditions such as joint reconstructive surgery.

### 2. Decision Aids, shared decision-making, and the Health Coach initiative

'Health Coaches' is an equity-oriented strategy designed to help disadvantaged populations to become better decision makers, negotiators, and navigators of the health system for their own health benefit. In a CIDA funded project in Chile, in partnership with the Pontifica Universidad Catolica de Chile (PUC) School of Nursing and the municipality of La Pintana, a proof-of-concept decision support program for disadvantaged women is being implemented to enhance women's evidence-based decision-making capacity in managing their own health and the health of their families [9]. Nursing students, primary care health professionals and individuals from the community receive training in 'coaching' rather than 'advising' to develop decision-making capacities rather than to create dependant relationships. Health coaches have access to evidence-based health information and decision aids. They provide information, clarify values, and develop skills in deliberation, communication, and behaviour change. Delivery of coaching and decision support can take the form of self-care manuals, online health information and decision aids, individual and group patient education and coaching sessions, skills training of primary care professionals, and population-based telephone call centres (Table 2). This program has recently received funding from a technology-transfer funder in Chile (FONDEF) to develop a call-centre in collaboration with a large telecommunications company in Chile.

**Table 2:** Health Coaching Decisions, Roles and Interventions

**Types of Decisions**	**Health Coach Role**	**Delivery Strategies & Resources**
Self-care and triage to appropriate level of professional care	**Approach**: 'Coaching' to develop self-confidence & skills 'Active' listening and feedback	• Health coaching services ○ in person ○ group ○ telephone call center
Chronic condition management (e.g. congestive heart failure, coronary heart disease, asthma, chronic obstructive pulmonary disease, diabetes, arthritis and chronic low-back pain)	**Process**: 1. Assess Decision Making Needs Strengths and deficits in knowledge, values clarity, skills, support, resources	• Online decision support worksheets to capture processes of decision support
Preference-sensitive health care options and related social decisions (e.g. menopause options, abnormal uterine bleeding, home versus institutional care, end of life care, education, employment, housing arrangements, child care, retirement)	2. Address Decision Making Needs • Information • Values clarification • Support and Skills Development: problem solving, decision making, communication, behaviour change, navigation to support and resources	• Online library of information, decision aids, and interventions • Online training programs ○ professional schools ○ high schools
	3. Facilitate progress in decision making and implementation	
	4. Facilitate transfer of learning to future decisions	

Potential decision making capacity development applications include:

• An online library of evidence-based information and decision aids as resources for the women and girls and individuals who provide information to them (health professionals, teachers, journalists, librarians);

• Online training programs to develop decision making skills in high schools;

• Online training programs for professionals in the health coach role (nursing, library science, education, journalism);

• Models of health coaching services, delivered in person, in groups, or via telephone call centers; and

• Online decision support worksheets to structure, capture data, and feed back information on participants' baseline decision-making needs, interventions provided, and progress in decision making.

**Policy-makers can access a generic decision aid at the Ottawa Patient Decision Aids Website **[10]**as well as a compendium of quality-assessed, evidence-based decision aids **[11].

### 3. CIET cycles

**Figure 1:**
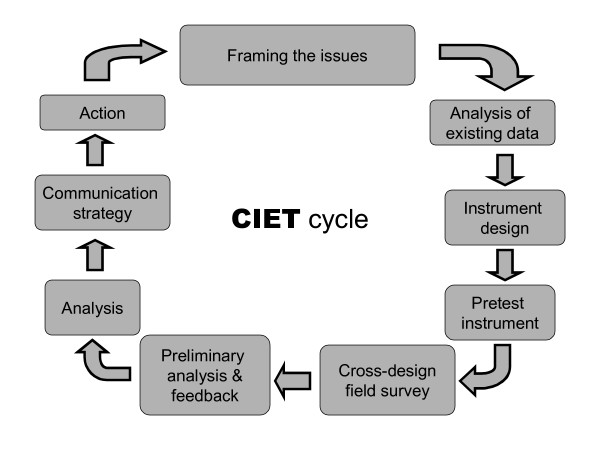
Evidence-based planning with the CIET Cycles: building capacity with successive Cycles.

The CIET cycles and methods are a tool used to support local evidence-based planning, that is available from CIET [12]. In the 1980s, at least partly in response to the difficulties being experienced in setting priorities and acting to achieve them, CIET developed its population-based applications of modern epidemiology in health planning [13]. Combining an adapted cluster survey technique with qualitative methods for discussing evidence with communities and health workers, the CIET methods are intended to support evidence-based decision making at local and national level. Buy-in from stakeholders centred on the formulation of what to measure (impact, coverage and costs). Consistent with the Triple-A approach, it introduced reiterative cycles of uptake and sharing of evidence, supplemented by a capacity building approach including transfer of measurement skills and community capacity building (Figure 1). This approach introduced formal epidemiological analysis to identify actionable risk and resilience factors, and also incorporated the community voice in a structured way, through focus groups and community meetings. The 1990s saw CIET methods applied in 47 countries worldwide, addressing issues such as access to health care services, gender gap in education, food security, prevention of sexual violence and corruption. CIDA supports application of the CIET methods in Pakistan as a governance support mechanism, emphasizing the community building and equity implications of the methods.

**Figure 2:**
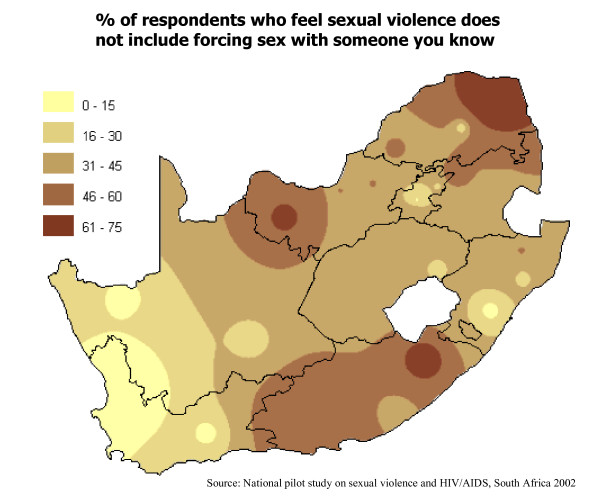
Example of a CIET map.

The popularization of geographic information systems (GIS) opens a new horizon for evidence-based health planning. More complex data can be portrayed attractively and, as a consequence, more people can participate in evidence-based decision making. Planners need to identify the mix of circumstances under which a health intervention is effective, to quantify the gaps between the intended and the actual, and to present alternatives for closing them. CIETmap is a free geomatics and epidemiology software developed by the CIET group [14]. Figure 2** shows an application of the CIET map tool to illustrate geographic differences in responses to questions regarding sexual violence and HIV/AIDS in South Africa. **The push-button mapping and analysis software allows users to generate maps as evidence-based decision aids directly from survey or routine institutional data. It combines raster and vector mapping techniques with epidemiological analysis tools. While no hardware or software can replace a solid practical training in epidemiology – and no technical training can replace a commitment to equity – customized epidemiological mapping software can provide an important tool for studying and comparing health indicators among and between different population groups.

CIET runs regular international training courses in the use of CIET methods. Interested users can inquire about these courses and training materials by contacting CIET International [15].

### 4. Ottawa Equity Gauge

Community-level action may be a very effective way of reducing health inequalities. Communities have the power and freedom to identify priority health problems, and to use a phased approach to modify the cultural, political, economic and social context in order to effect change.

**Figure 3:**
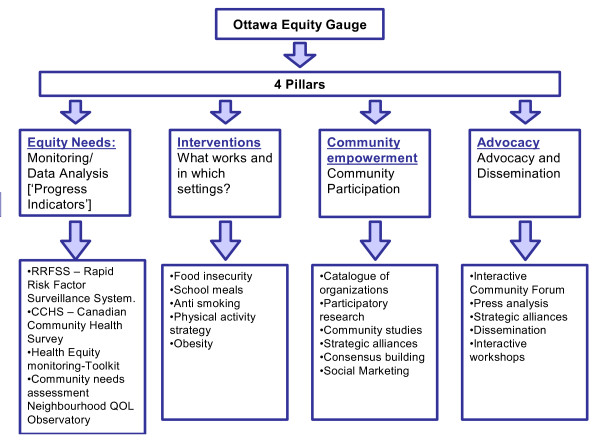
Ottawa Equity Gauge.

The Ottawa Equity Gauge project will measure, monitor and address health inequities in accidents, exercise, nutrition and smoking in Ottawa. It is based on the Global Equity Gauge Alliance's framework that has been implemented in 11 low and middle-income countries since 2000 [16]. This framework seeks to reduce health inequities through three broad spheres of action, described as pillars. These pillars are (Figure 3); 1)Assessment and Monitoring, 2)Advocacy, and 3)Community Empowerment. The Ottawa Equity gauge also emphasizes a fourth 'Intervention" pillar based upon Cochrane and Campbell systematic reviews of the interventions. The Ottawa Equity Gauge project brings together researchers, community leaders, and stakeholders. The current focus is on identifying food security and nutrition issues in Ottawa using a mix of participatory action research and systematic reviews of published and unpublished literature. Partners include representatives from non-governmental organizations (eg Ottawa Just Food, Centretown Community Health Centre) as well as policy-makers (City of Ottawa Health Department) and multiple disciplines from the University of Ottawa. The Ottawa Equity Gauge team has recently completed a survey to understand food insecurity in vulnerable populations in Ottawa that is now being used for advocacy. The Ottawa Equity Gauge team is now assessing spatial inequalities in food insecurity, with funding from the Canadian Institutes of Health Research. The Ottawa Equity Gauge has recently been approved as a GEGA associate and has recently co-supervised a Canadian Society of International Health Intern with Antoinette Ntuli of GEGA. The Global Equity Gauge Alliance has reference material on how to set up an Equity Gauge [17].

The **Measurement and monitoring **pillar involves the analysis, description and understanding of health inequities through the collection and collation of qualitative and quantitative data information [16]. This will identify targets for action and enable later evaluation on whether change has occurred. Some possible sources for data have already been selected including the Ottawa subsets of the 2001/02 Canadian Community Health Survey, the 1996/97 National Population Health Survey, the Canadian Fitness and Lifestyle Research Institute Survey, and the Ottawa Rapid Risk Factor Surveillance System. The **Intervention **pillar aims to address the lack of evidence that exists on the effectiveness of interventions to reduce health disparities through the accumulation and synthesis of research. Most notably this will involve systematically reviewing the evidence for public health interventions on low socio-economic groups, rather than just that of population averages. The information gained on the effectiveness of interventions as well as the data gathered from the monitoring pillar will improve the capacity for the promotion of pro-equity policies. Advocacy will be combined with a bottom up approach **of community empowerment and capacity building. **This pillar places importance on working with communities to help them speak more effectively for themselves, and building the capacity of individuals and community groups to improve their own health. An Equity Gauge therefore does not merely describe health disparities but rather couples data collection with coordinated community-driven actions and advocacy efforts to reduce disparities and help members of the community to reach their full health potential.

### 5. The needs-based health assessment toolkit

This toolkit was developed in response to the recommendations from the Ottawa Conference on Exploring Global Interfaces [18]. The Toolkit represents a valuable synthesis of methods to assemble the information on which clinical and health policy decisions about technologies can be based. The Toolkit uses the Technology Assessment Iterative Loop (TAIL) as an overall framework [19] & is accessible on the internet [20].

The toolkit focuses on choosing health interventions based on the health needs of a population, using an iterative approach. The iterative steps are: 1) Health needs assessment; 2) Priority setting and needs-based technology assessment; 3) Community effectiveness; 4) Cost-effectiveness; and 5) Policy, strategy and management. Step 6 involves re-iterating back to needs assessment to monitor the impact of needs-based health technology assessment. The tools in the five steps involve the input from many disciplines, for example; social scientists, health care professionals, biostatisticians, stakeholders and consumers, policy makers and computer specialists. This toolkit has been used as a training tool by the WHO Collaborating Centre for Health Technology Assessment. Furthermore, international research fellows and research interns have worked on chapters of the toolkit as part of their training. The toolkit has been widely disseminated at meetings including ISTAHC, the Global Forum, the Cochrane Collaboration and PAHO conferences.

The tool kit has focused on averages, but is now being expanded to include the above methods for assessing distributional issues so that equity gradients will be detected and included in any indicators. Peter Tugwell and colleagues have developed the equity-effectiveness loop framework to assess the "staircase effect" of reductions in efficacy in disadvantaged populations [21]. Its innovation will also lie in incorporating the new advances in knowledge translation (i.e. the development and evaluation of how these tools are being used and how to make these tools transferable). Case studies will be developed to describe successes and challenges in implementation [22].

#### Feedback from break-out groups

Suggested approaches to applying tools in diverse settings were proposed by participants in break-out groups:

• It is important to address both national and international levels of action. Some aspects of the transdisciplinary methods are universally applicable but at the national level, the political context and local history of health system reform needs to be taken into account.

• The process of arriving at consensus is as important as the conclusions. It is key to involve all the stakeholders and processes to make that happen, to acknowledge political will and balance (versus other priorities), involve communities in planning and identification of needs, and tailor the instruments to user needs.

Participants in the breakout groups identified several future research questions including:

1. What are the key factors that lead to the failure (and some successes) of previous initiatives?

2. What are the real determinants of equity and their impact (not just health)? How do we measure them?

3. What are the barriers to achieving equity (within and outside the health sector)?

4. How can cost effectiveness of interventions be used in decision-making such that economic efficiency decisions do not exacerbate inequities?

5. How do we manage competing agendas? What are the impacts of competing agendas?

6. Who are the key decision-makers and how do they, or will they use evidence? What is the impact of such a process?

7. What is the impact of a policy on widening or narrowing equity gap?

8. What impact has health reform had on Human Resources?

9. Have existing policies been implemented?

## Conclusion

Evidence-based planning practices have much to offer the goal of equitable health development. When captured well, evidence from communities can generate important and surprising insights. More fundamentally, the data generated can sometimes challenge the perceptions of those in authority and begin to change attitudes and agendas about equity.

Despite the technological advances that make evidence-based planning possible, many of the old questions still remain to be answered about how it is all to be done. Where should the evidence come from? How exactly are local decisions to be taken on the evidence? What are the precise mechanisms for sustained and meaningful community participation? What are the keys for starting a new cycle of assessment, analysis and action? How exactly do the changing health circumstances and changing health behaviors lead to different decisions, which then supposedly redirect and continue stimulating community participation?

Perhaps part of the answer lies in the detail. Just as important as the evidence collected is the process by which it is done. When the evidence generated is assimilated, interpreted and owned by the communities whose development it is meant to serve, evidence-based planning has the additional effect of creating an environment of sustained participation and transparency. If this dynamic can be activated, governments can acquire the skills to facilitate an evidence-driven and participatory process, and civil society groupings will become more able advocates for effecting change.

Different countries will want to use methods appropriate for the specific country with appropriate tailoring of the policy tool components, as described by some of the suggestions from the break-out groups.

## Abbreviations

CIDA – Canadian International Development Agency

CIET – Community Information and Epidemiological Technologies

ISTAHC – International Society of Technology in Health Care

PAHO – Pan American Health Organisation

PHC – Primary Health Care

RBM – Results Based Management

UNICEF – United Nations Children Fund

WHO – World Health Organisation

## Competing interests

The author(s) declare that they have no competing interests.

## Authors' contributions

PT had the idea for the manuscript and drafted the manuscript. AO, NA, SM, BK, MJ, VR, JHR, BS, GW, JL, DF participated in the writing and interpretation of data. All authors read and approved the final manuscript.
